# Population Survey Data Informing the Therapeutic Potential of Classic and Novel Phenethylamine, Tryptamine, and Lysergamide Psychedelics

**DOI:** 10.3389/fpsyt.2019.00896

**Published:** 2020-02-11

**Authors:** James D. Sexton, Charles D. Nichols, Peter S. Hendricks

**Affiliations:** ^1^ Department of Health Behavior, School of Public Health, University of Alabama at Birmingham, Birmingham, AL, United States; ^2^ Department of Pharmacology and Experimental Therapeutics, LSU Health Sciences Center, New Orleans, LA, United States

**Keywords:** phenethylamines, tryptamines, lysergamides, psychedelic-assisted therapy, mental health outcomes

## Abstract

**Introduction:**

The majority of contemporary psychedelic research has focused on ayahuasca, lysergic acid diethylamide, and psilocybin, though there are hundreds of novel psychedelic compounds that may have clinical utility. The purpose of the present study was to evaluate the therapeutic potential of classic and novel phenethylamine, tryptamine, and lysergamide psychedelics *via* a large, nationally representative population-based survey.

**Methods:**

We tested the unique associations of lifetime classic and novel phenethylamine, tryptamine, and lysergamide psychedelics with past month psychological distress and past year suicidality among respondents pooled from years 2008–2017 of the National Survey on Drug Use and Health (weighted N = 260,964,827).

**Results:**

Lifetime classic tryptamine use was associated with a decreased odds of past month psychological distress [aOR = 0.76; (0.69–0.83)] and past year suicidal thinking [aOR = 0.79; (0.72–0.87)]. Lifetime novel phenethylamine use, on the other hand, was associated with an increased odds of past year suicidal thinking [aOR = 1.44; (1.06–1.95)] and past year suicidal planning [aOR = 1.60; (1.06–2.41)]. No other significant associations were found.

**Discussion and Conclusions:**

These findings, which may be driven by differences in pharmacodynamics, suggest that classic tryptamines may hold the greatest therapeutic potential of the psychedelics, whereas novel phenethylamines may pose risk for harm. The present findings thus support continued research on the clinical application of classic tryptamines. Though the current results caution against the clinical utility of novel phenethylamines, further study of these and other novel psychedelic substances is nonetheless warranted to better understand their potential application.

## Introduction 

Classic psychedelics, which include dimethyltryptamine (DMT), lysergic acid diethylamide (LSD), mescaline, and psilocybin, have been studied clinically, anthropologically, and sociologically (1, 2). Classic psychedelics appear to be both generally safe and potentially therapeutic in the treatment of anxiety disorders, mood disorders, and substance use disorders (3–7). Consistent with findings from clinical trials, population-level analyses demonstrate that lifetime classic psychedelic use is associated with a reduced likelihood of past month psychological distress and past year suicidality (8). Lifetime psilocybin use in particular evinced these protective associations above and beyond other lifetime classic psychedelic use in one analysis, suggesting that psilocybin may have unique therapeutic potential (9), however, this analysis collapsed all non-psilocybin classic psychedelics across the three primary categories of classic psychedelics: phenethylamines (mescaline and the mescaline-containing cacti peyote and San Pedro), tryptamines (DMT and the DMT-containing admixture ayahuasca; psilocybin is also a tryptamine), and lysergamides (LSD). Whether the unique protective associations of psilocybin apply to all tryptamines, and whether tryptamines in general may have unique therapeutic potential relative to phenethylamines and lysergamides is unknown.

Novel psychedelics, which also comprise phenethylamines, tryptamines, and lysergamides, are distinct from classic psychedelics in that they lack both the long history of human use and substantial research data investigating their general safety, though there are notable pharmacologic and chemical data on these substances (10–13). From 2005 to 2017, novel phenethylamines (i.e. 2,5-Dimethoxy-4-"X"-phenethylamine or 2C-X, N-Benzyl Derivatives or NBOME’s) accounted for the majority of novel drug mentions in the National Survey on Drug Use and Health (NSDUH) (14), suggesting naturalistic use of these substances is on the rise. One population-level analysis found that lifetime novel psychedelic use is rare, accounted for primarily by phenethylamines, and associated with an increased likelihood of past month psychological distress and past year suicidality relative to lifetime use of classic psychedelics only (15). This suggests that novel psychedelics may be distinct from and carry reduced therapeutic potential relative to classic psychedelics. However, as with the abovementioned analysis, this analysis collapsed all classic psychedelics across phenethylamines, tryptamines, and lysergamides, and collapsed all novel psychedelics across a variety of subcategories, potentially obscuring any meaningful differences between the three primary categories of novel psychedelics. Whether each of the three categories of novel psychedelics may be distinct from and carry reduced therapeutic potential relative to each of the three categories of classic psychedelics is unknown.

Exploring the therapeutic potential of classic and novel phenethylamine, tryptamine, and lysergamide psychedelics is relevant considering that psychedelic research is experiencing a modest but growing resurgence. Whereas almost all contemporary research is accounted for by ayahuasca, LSD, and psilocybin (16), there are hundreds of novel psychedelic compounds that might have clinical utility (17, 18), with population-based survey respondents reporting the use of over 40 such compounds (15). Winnowing down this extensive list of psychedelic substances to those most likely to carry therapeutic benefit would help direct future study. Though classic and novel phenethylamine, tryptamine, and lysergamide psychedelics share important similarities (e.g. 5-HT2A receptor agonism), they differ in chemical structure, which appears to account for differences in reported subjective effects (19). It is known that psychedelics interact differently with their target 5-HT_2A_ receptor (20). That is to say, they engage with different sets of amino acid residues in the binding pocket of the receptor to produce slightly different active state conformations of the receptor. The differences in conformational states lead to known differential or biased recruitment of second messenger and effector pathways that ultimately alter the physiology of the cell or neuron such that how the classic lysergamide psychedelic LSD alters cellular physiology is slightly different from how the classic phenethylamine psychedelic mescaline does. Indeed, it is has been hypothesized that these functional differences in receptor/ligand interactions and differential effects on cellular physiology are linked to their respective subjective experiences (21).

The purpose of this study was to test for unique associations of lifetime use of classic and novel phenethylamines, tryptamines, and lysergamide psychedelics with mental health outcomes using data from a large, nationally representative population-based survey. Considering the regulatory and other complexities associated with administering psychedelic substances to humans, population-based surveys represent useful springboards for exploring the therapeutic potential of these compounds (8). Thus, the present analysis will provide preliminary evidence with regard to which categories of classic and novel psychedelics might hold the greatest therapeutic potential, thereby informing future clinical research.

## Methods

### Data

Data were obtained from the publicly available NSDUH, a survey of the general, non-institutionalized United States population aged 12 and older administered by the Substance Abuse and Mental Health Services Administration of the US Department of Health and Human Services. The survey uses a multistage probability sampling design where individuals are randomly selected within a roster that accounts for state population size and housing inventory. NSDUH interviewers met with respondents in their homes, who listened to pre-recorded interview guides on headphones and responded via computer prompt. We combined the data from 2008–2017 in order to maximize sample size while maintaining standardized assessment procedures introduced in 2008. The comprehensive NSDUH sampling and questionnaire methodology can be found on their website https://nsduhweb.rti.org/respweb/about_nsduh.html.

### Respondents

Using SPSS syntax, individual respondents from the 2008–2017 NSDUH were given a unique identifier and combined into a single database using the Cantor pairing function for a total unweighted sample of 562,072 cases. The analytic sample included all respondents with valid responses to the primary and secondary variables, yielding a total unweighted sample size of 354,535 (see **Supplementary Table 1** for psychosocial characteristics of the sample). The *Analysis* section includes sample sizes for each regression model as the sample sizes varied based upon the dependent variable used. Respondents reporting mescaline (MESC2 = 1 and code 603 from variables HALNEWA, HALNEWB, HALNEWC, HALNEWD, HALNEWE = 1), peyote or San Pedro (cacti that contains mescaline; PEYOTE2 = 1 and code 602 and 6077 respectively from variables HALNEWA, HALNEWB, HALNEWC, HALNEWD, HALNEWE = 1), were coded as positive for lifetime classic phenethylamine use. Respondents reporting they had ever, even once used DMT (code 616 from variables HALNEWA, HALNEWB, HALNEWC, HALNEWD, HALNEWE = 1), ayahuasca (an admixture that contains DMT; code 6103 from variables HALNEWA, HALNEWB, HALNEWC, HALNEWD, HALNEWE = 1), or psilocybin (PSILCY2 = 1 and code 604 from variables HALNEWA, HALNEWB, HALNEWC, HALNEWD, HALNEWE = 1) were coded as positive for lifetime classic tryptamine use. Respondents who reported using LSD (LSDFLAG = 1, and code 601 from variables HALNEWA, HALNEWB, HALNEWC, HALNEWD, HALNEWE = 1) were coded positive for lifetime classic lysergamide use, whereas those reporting they had never used any of the aforementioned substances were coded as negative for each respective drug category (8, 9, 15). Respondents were given the option to write-in other “hallucinogens” they had used, and novel psychedelics were gathered from write-in responses as per Sexton et al. (15). **Table 1** lists both classic and novel psychedelic compounds and their classification for the purposes of this analysis. Respondents who indicated they had ever taken a substance that was classified as a novel phenethylamine (code in **Table 1** from variables HALNEWA, HALNEWB, HALNEWC, HALNEWD, HALNEWE = 1) were coded as positive for lifetime novel phenethylamine use. Respondents who indicated they had ever taken a substance that was classified as a novel tryptamine (code in **Table 1** from variables HALNEWA, HALNEWB, HALNEWC, HALNEWD, HALNEWE = 1) were coded as positive for lifetime novel tryptamine use. Respondents who indicated they had ever taken a substance that was classified as a novel lysergamide (code in **Table 1** from variables HALNEWA, HALNEWB, HALNEWC, HALNEWD, HALNEWE = 1) were coded as positive for lifetime novel lysergamide use, whereas those reporting they had never used novel phenethylamines, tryptamines, or lysergamides were coded as negative for lifetime use of those respective compounds. Respondents who responded to the write-in query with “no” and those who did not provide a write-in a response were coded as negative for each of the novel psychedelic use variables. **Supplementary Table 2** presents correlations among lifetime classic and novel phenethylamine, tryptamine, and lysergamide use. It is noted that these correlations ranged from very modest (e.g., lifetime classic phenethylamine use with lifetime novel lysergamide use) to moderate (lifetime classic phenethylamine use with lifetime classic tryptamine use and lifetime classic lysergamide use) to strong (lifetime classic tryptamine use with lifetime classic lysergamide use).

**Table 1:** Psychedelic compounds reported by respondents from the 2008–2017 National Survey on Drug Use and Health (NSDUH), respective NSDUH codes, and citations to supporting literature.

**Classic Phenethylamines**	**Novel Phenethylamines (continued)**	**Novel Trypamines (continued)**
Peyote (code 602; variable PEYOTE2)	NBOMe: Otherwise Unspecified (code 6203) (13)	4-AcO-DiPT (code 6177) (22)
San Pedro (code 6077)	TCB-2 (code 6180) (23)	4-AcO-DMT (code 6171, 6178) (24)
Mescaline (code 603; variable MESC)	Bromo-DragonFly (code 6176) (25)	4-AcO-MET (code 6202) (26)
**Novel Phenethylamines**	DOC (code 6169) (27)	5-MeO-DALT (code 6183) (28)
2C-B (code 698) (29)	DOB (code 6173) (30)	5-MeO-DiPT (code 6130) (30)
2C-C (code 6197, 6139) (31)	DOI (code 6168) (30)	5-MeO-DMT (code 6061) (32)
2C-D (code 6154) (31)	DOM (code 636) (32)	5-MeO-MiPT (code 6192) (30)
2C-E (code 6138) (31)	**Classic Tryptamines**	5-MeO: Otherwise Unspecified (code 6146) (33)
2C-I (code 6126) (31)	Psilocybin (code 604; variable PSILCY2)	**Classic Lysergamides**
2C-P (code 6182) (29)	DMT (code 616)	LSD (code 601; variable LSDFLAG)
2C-T-2 (code 6112) (31)	Ayahuasca (code 6103)	
2C-T-7 (code 6100) (29)	**Novel Tryptamines**	**Novel Lysergamides**
2C-T-21(code 6172) (35)	DPT (code 6141) (36)	1P-LSD (code 6209) (34)
2C-x (code 6143) (29)	DiPT (code 6144) (30)	LSZ (code 6195) (37)
2C-T (code 6159) (35)	MiPT (code 6140) (38)	AL-LAD (code 6200) (34)
2C-F (code 6190) (35)	4-HO-DET (code 6201) (39)	ALD-52 (code 652) (40)
25i-NBOMe (code 6185) (41)	4-HO-DiPT (code 6175) (42)	
25b-NBOMe (code 6188) (13)	4-HO-MET (code 6181) (42)	
25c-NBOMe (code 6189) (13)	4-HO-MiPT (code 6179) (38)	

### Analysis

Four multivariate logistic regression models were created to test the associations of 1) past month psychological distress (unweighted n = 356,046; variable SPDMON; yes = 1 or no = 0) as measured by the widely-used and well-validated six-item Kessler Psychological Distress Scale (K6; consistent with K6 scoring guidelines and its application in research, the NSDUH uses a dichotomous cutoff score ≥13; 43, 44), 2) past year suicidal thinking (unweighted n = 354,580; “At any time in the past 12 months … did you seriously think about trying to kill yourself? ”; variable MHSUITHK; yes = 1 or no = 0), 3) past year suicidal planning (unweighted n = 354,555; “During the past 12 months, did you make any plans to kill yourself? ”; variable MHSUITRY; yes = 1 or no = 0), and 4) past year suicide attempt (unweighted n = 354,552; “During the last 12 months, did you try to kill yourself? ”; variable MHSUITRY; yes = 1 or no = 0) with the following independent variables: lifetime use of classic phenethylamines (yes = 1 or no = 0), lifetime use of classic tryptamines (yes = 1 or no = 0), lifetime use of classic lysergamides (yes = 1 or no = 0), lifetime use of novel phenethylamines (yes = 1 or no = 0), lifetime use of novel tryptamines (yes = 1 or no = 0), and lifetime use of novel lysergamides (yes = 1 or no = 0; all independent variables were entered simultaneously). Consistent with prior analyses making use of NSDUH data (8, 15), the following covariates were included in the regression models to control for potential sources of confounding: age in years (12–17, 18–25, 26–34, 35–49, 50–64, or 65 or older); sex (male or female); ethnoracial identity (non-Hispanic White, non-Hispanic African American, non-Hispanic Native American/Alaska Native, non-Hispanic Native Hawaiian/Pacific Islander, non-Hispanic Asian, non-Hispanic more than one race, or Hispanic); educational attainment (5th grade or less, 6th grade, 7th grade, 8th grade, 9th grade, 10th grade, 11th grade, 12th grade, freshman college year, sophomore or junior college year, or senior college year or more); annual household income (less than $20,000, $20,000–$49,999, $50,000–$74,999, or $75,000 or more); marital status (married, divorced/separated, widowed, or never married); self-reported engagement in risky behavior (“How often do you like to test yourself by doing something a little risky? ”; never, seldom, sometimes, or always); and lifetime use of cocaine, other stimulants, sedatives, tranquilizers, heroin, pain relievers, marijuana, phencyclidine, 3,4-methylenedioxymethamphetamine (MDMA/ecstasy), and inhalants (each aforementioned drug category coded as separate covariates). Logistic regression models were created in R version 3.5.1 using the package “survey” and the svydesign and svyglm functions to account for the complex survey design used by the NSDUH (45, 46), and the package “jtools” to generate 95% confidence intervals and adjusted odds rations for each model (47). Lifetime novel lysergamide use, though quite rare (N = 9 unweighted respondents) was included in the regression models despite the fact that all novel lysergamide users also reported classic lysergamide use. Despite this overlap, multi-collinearity was not present within the model. However, associations of lifetime novel lysergamide use are not reported here given difficulty in interpretation. Indeed, adjusted ORs (all non-significant) revealed values well outside the range of all other variables included in regression models. All of the SPSS syntax, R source code, and datasets used to conduct these analyses are hosted on the Open Science Framework at the following link https://osf.io/xgqmd/.

## Results

The weighted frequency of lifetime use of each psychedelic category and lifetime use of specific substances within each of these categories can be found in **Table 2**. As shown in this table, lifetime use of classic psychedelics was much more common than lifetime use of novel psychedelics. Lysergamides were the most commonly used category of classic psychedelic with approximately 10% of the United States population reporting lifetime use, whereas phenethylamines were the most commonly used category of novel psychedelic with one-tenth of one percent of the United States population reporting lifetime use. Psilocybin accounted for the vast majority of those reporting lifetime classic tryptamine use.

**Table 2:** Weighted frequencies of lifetime use of each psychedelic category and lifetime use of specific substances within each of these categories from the 2008–2017 NSDUH.

**Classic Phenethylamines** *(10,332,715; 4.0%)*	**Novel Phenethylamines (continued)**	**Novel Tryptamines (continued)**
		5-MeO-DALT (530; 0.0002%)
Peyote (5,619,308; 2.2%)	DOC (4,994; 0.002%)	5-MeO-DiPT (2,544; 0.001%)
San Pedro (13,513; 0.005%)	DOB (5,181; 0.002%)	5-MeO-DMT (7,889; 0.003%)
Mescaline (8,158,409; 3.1%)	DOI (1,549; 0.0006%)	5-MeO-MiPT (9,383; 0.004%)
**Novel Phenethylamines**	DOM (16,630; 0.006%)	5-MeO: OU (2,392; 0.0009%)
(277,683; *0.1%)*	**Classic Tryptamines**	**Classic Lysergamides**
2C-B (119,206; 0.05%)	*(22,077,615; 8.5%)*	*(24,664,123; 9.5%)*
2C-C (876; 0.0003)	Psilocybin (22,053,740; 8.5%)	LSD
2C-D (406; 0.0002%)	DMT (252,452; 0.1%)	
2C-E (58,969; 0.02%)	Ayahuasca (52,122; 0.02%)	**Novel Lysergamides**
2C-I (99,203; 0.04%)		*(2,237; 0.0009%)*
2C-P (10,030; 0.004%)	**Novel Tryptamines**	
2C-T-2 (5,158; 0.002%)	*(30,835; 0.01%)*	1P-LSD (153; 0.00006%)
2C-T-7 (7,319; 0.003%)		LSZ (1,370; 0.0005%)
2C-T-21 (1,290; 0.0005%)	DPT (455; 0.0002%)	AL-LAD (248; 0.0001%)
2C-X (0; 0.0%)	DiPT (166; 0.00006%)	ALD-52 (466; 0.0002%)
2C-T (1,400; 0.0005%)	MiPT (0; 0.0%)	
2C-F (124; 0.00005%)	4-HO-DET (1,495; 0.0006%)	
25i-NBOMe (27,020; 0.01%)	4-HO-DiPT (513; 0.0002%)	
25b-NBOMe (2,878; 0.001%)	4-HO-MET (930; 0.0004%)	
25c-NBOMe (4,827; 0.002%)	4-HO-MiPT (357; 0.0001%)	
NBOMe: OU (3,124; 0.001%)	4-AcO-DiPT (0; 0.0%)	
TCB-2 (1,956; 0.0008%)	4-AcO-DMT (7,141; 0.003%)	
Bromo-DragonFly (1,598; 0.0006%)	4-AcO-MET (252; 0.0001%)	
		

Frequencies reported here are formatted as such: (weighted N’s; weighted %’s of total US population; OU, Otherwise Unspecified).

**Figure 1:**
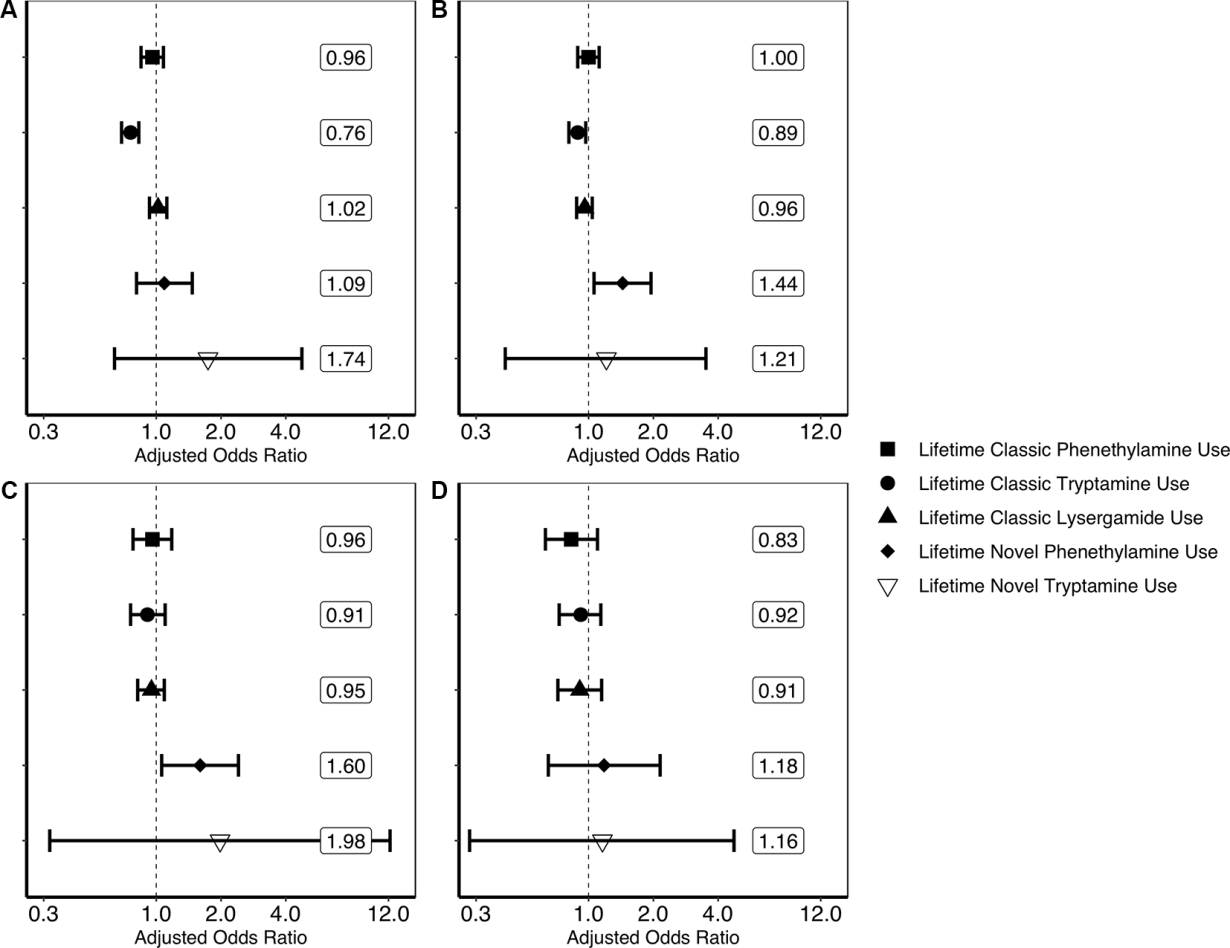
Results of multivariate logistic regression models predicting past month psychological distress and past year suicidality. **(A)** Result of multivariate logistic regression model predicting past month psychological distress (unweighted *n* = 356,046). **(B)** Result of multivariate logistic regression model predicting past year suicidal thinking (unweighted *n* = 354,580). **(C)** Result of multivariate logistic regression model predicting past year suicidal planning (unweighted *n* = 354,555). **(D)** Result of multivariate logistic regression model predicting past year suicide attempt (unweighted *n* = 354,552). Each plotted shape relates to the drug category and represent weighted adjusted odds ratio point estimates and error bars are 95% confidence intervals. Associations are adjusted for the following covariates: age in years (12–17, 18–25, 26–34, 35–49, 50–64, or 65 or older); sex (male or female); ethnoracial identity (non-Hispanic White, non-Hispanic African American, non-Hispanic Native American/Alaska Native, non-Hispanic Native Hawaiian/Pacific Islander, non-Hispanic Asian, non-Hispanic more than one race, or Hispanic); educational attainment (5th grade or less, 6th grade, 7th grade, 8th grade, 9th grade, 10th grade, 11th grade, 12th grade, freshman college year, sophomore or junior college year, or senior college year or more); annual household income (less than $20,000, $20,000–$49,999, $50,000–$74,999, or $75,000 or more); marital status (married, divorced/separated, widowed, or never married); self-reported engagement in risky behavior (“How often do you like to test yourself by doing something a little risky?”; never, seldom, sometimes, or always); and lifetime use of cocaine, other stimulants, sedatives, tranquilizers, heroin, pain relievers, marijuana, phencyclidine (PCP), 3,4-methylenedioxymethamphetamine (MDMA/ecstasy), and inhalants (each aforementioned drug category coded as separate covariates). Associations of covariates with psychological distress and suicidality are not reported here. The associations of lifetime novel lysergamide use are not evaluated here as noted in the *Discussion*.

Findings generated from the four multivariate logistic regression models can be seen in **Figure 1**. These models show that lifetime classic tryptamine use was associated with a decreased odds of past month psychological distress [adjusted odds ratio or aOR = 0.76; (0.69–0.83)] and past year suicidal thinking [aOR = 0.79; (0.72–0.87)]. Novel phenethylamine use, however, was associated with an increased odds of past year suicidal thinking [aOR = 1.44; (1.06–1.95)] and past year suicidal planning [aOR = 1.60; (1.06–2.41)]. No other significant associations were found.

## Discussion

The objective of the present analysis was to test unique population-level associations of classic and novel phenethylamine, tryptamine, and lysergamide use with psychological distress and suicidality, thereby providing one line of evidence regarding which categories of psychedelics might hold the greatest therapeutic potential. We found that lifetime classic tryptamine use, the vast majority of which was accounted for by psilocybin, was associated with a reduced likelihood of past month psychological distress and past year suicidal thinking above and beyond a range of covariates including lifetime use of other classic psychedelics and lifetime use of novel psychedelics. These findings are consistent with a prior analysis indicating that lifetime psilocybin use may be especially protective against psychological distress and suicidality as compared to other classic psychedelics (9). Results were also consistent with a number of recent clinical trials suggesting that psilocybin is a promising therapeutic agent for end-of-life anxiety, treatment-resistant depression, alcohol dependence, and tobacco dependence (3, 4, 48–50). It is noted that though very few respondents reported lifetime use of ayahuasca, recent clinical trials suggest a substantial and rapid antidepressant effect of this DMT-containing admixture (51, 52). It may be, therefore, that classic tryptamines are among the most promising therapeutic agents of the psychedelics.

Sexton et al. found that lifetime use of novel psychedelics increased the likelihood of past year suicidal thinking and planning compared to lifetime classic psychedelic use only (15). In the present study, we found that novel phenethylamine use was associated with an increased likelihood of past year suicidal thinking and planning above and beyond several covariates including lifetime use of classic psychedelics and lifetime use of other novel psychedelics. Lifetime use of novel tryptamines was not associated with psychological distress or suicidality. The same was true of novel lysergamides, though interpretation of this finding is complicated by very few respondents reporting the use of novel lysergamides and the fact that all novel lysergamide users also reported the use of classic lysergamides. Nevertheless, this suggests that novel phenethylamine use accounts for the prior associations of Sexton et al., and that novel phenethylamines may be, to some degree, potentially harmful to mental health (15). Indeed, there have been a number of adverse event reports from novel phenethylamine use including psychosis, neurovascular hemorrhages, and seizures (53–56). These findings support the conclusion that novel phenethylamine psychedelics may be distinct from other psychedelic categories in that they may confer harm.

Tryptamine-based compounds in general have affinity for and agonist activity at primarily several different serotonin receptors. For example, psilocin, the active metabolite of the prodrug classic tryptamine psychedelic psilocybin, has varying but appreciable affinity for all serotonin receptors, with the exception of the 5-HT_3_ receptor, where it acts as an agonist, and the 5-HT_7_ receptor, where it is an antagonist. Significantly, all known tryptamines that have been tested have affinity for and agonist activity at 5-HT_1A_ receptors. Activation of this receptor has been associated with antidepressant activity, and proposed as an important mechanism of the antidepressant effects of selective serotonin reuptake inhibitor medications (57, 58). Indeed, new antidepressant medications on the market were specifically designed to have at least partial agonist activity at 5-HT_1A_ receptors (59). It is possible that activation of 5-HT_1A_ receptors within the brain by classic tryptamine psychedelics confers positive effects to affective states and the observed reduction of psychological distress and suicidality in users. This may also apply to novel tryptamine psychedelics, though lifetime use of novel tryptamine psychedelics was not associated with psychological distress or suicidality in the current study, perhaps due to a lack of statistical power.

The phenethylamine compounds listed in **Table 2**, especially the novel phenethylamine 2C class, more often have affinity for and activity at the alpha-adrenergic receptor as well as moderate affinity for blockade of norepinephrine and dopamine transporters, whereas most tryptamines do not (60–63). Further, there is little to no activation of 5-HT_1A_ receptors by these drugs. Together, activation of alpha adrenergic receptors with increases in synaptic norepinephrine and dopamine would be predicted to induce behavioral outcomes similar to amphetamines, including negative effects on cognitive behavioral control (64). These pharmacological outcomes, predicted to occur more frequently with phenethylamine (and especially the novel 2C phenethylamine) drugs than tryptamines, could underlie the observed associations of these novel phenethylamines with negative psychological health. In support of this view, 2C-B, the most commonly reported novel phenethylamine, is often substituted for MDMA among electronic music party goers secondary to its purported psychostimulant properties (15, 20, 65). Indeed, novel phenethylamines are often described in terms of psychostimulant effects (20, 29), whereas challenging, emotional breakthrough, and mystical-type experiences appear to underlie the therapeutic outcomes of the classic tryptamine psychedelic psilocybin (16, 66, 67). Thus, with regard to acute subjective effects, it may be that novel phenethylamines are characterized more so by problematic psychostimulant outcomes and less so by salubrious challenging, emotional breakthrough, and mystical-type experiences. It is important to interpret these associations with caution, however, as the NSDUH only provides data on naturalistic psychedelic use and it is quite possible that certain novel phenethylamines hold therapeutic potential when administered in a controlled environment.

A strength of the current study includes the assessment of a large, nationally representative sample of respondents from real-world settings. Additionally, the code used to conduct these analyses and the data sets that were analyzed are freely available online on the Open Science Framework. As in prior analyses, this analysis used a range of covariates to control for a number of sources of confounding (8, 9, 15). Furthermore, when estimating the associations of one independent variable (e.g., lifetime classic tryptamine use), our models controlled for the other five independent variables (e.g., lifetime classic phenethylamine use, lifetime classic lysergamide use, lifetime novel phenethylamine use, lifetime novel tryptamine use, and lifetime novel lysergamide use). Despite this approach, a number of limitations should be noted. First, an obvious limitation is reliance on self-report, which may have obfuscated true relationships between classic and novel psychedelic use and mental health outcomes. Second, as with any population-based survey, we could not control for every possible source of confounding. Any number of unassessed covariates may account for the associations reported here. For instance, perhaps classic tryptamine users are especially open to new experience and spiritual, and therefore the reported associations reflect the influence of these traits, rather than an effect of classic tryptamine use. Moreover, novel phenethylamine users may be especially prone to neuroticism, and therefore associations with suicidal thinking and planning may capture the impact of this characteristic on these outcomes (see 8). As noted above, the novel phenethylamine 2C-B may have a reputation as a “party drug, ” and thus the associations reported here may reflect the influence of recreational use motives. One such motive may be sensation seeking (see 68–71) which can be defined as a trait characterized by “the seeking of varied, novel, complex, and intense sensations and experiences, and the willingness to take physical, social, legal, and financial risks for the sake of such experience” (72, page 27). Though the inclusion of self-reported engagement in risky behavior as a covariate in analyses likely accounted for some of the variance in this trait, sensation seeking itself, in addition to a number of other relevant psychological constructs (e.g., openness and neuroticism), was not assessed by the NSDUH. In any event, as with any cross-sectional survey, the present results may not necessarily indicate causation. Third, as analyses were restricted to the available data (i.e., whether or not a respondent had used a classic or novel phenethylamine, tryptamine, or lysergamide psychedelic in his or her lifetime), dose-response relationships as well as associations with frequency of use, age of first use, recency of use, and any number of other variables pertaining to use patterns could not be tested. Future surveys including the NSDUH that seek to better understand the relationships of psychedelic use with mental health would benefit from the assessment of more complex use patterns rather than simple lifetime use. Additionally, there was overlap among lifetime classic and novel phenethylamine, tryptamine, and lysergamide psychedelic use, which might have limited the ability to detect the unique associations of these predictor variables with the outcomes (e.g., lifetime classic lysergamide use might be associated with a reduced likelihood of psychological distress and suicidality, but not above and beyond lifetime classic tryptamine use, with which it was strongly correlated). Fourth, population-level associations may obscure effects at the individual level. Thus, despite the reported trends, it is possible that some individuals were harmed by classic tryptamine use, whereas others benefited from novel phenethylamine use. Finally, as noted in Sexton et al., the write-in nature of lifetime novel psychedelic use likely lead to underreporting of these substances, which potentially affected the current estimates, including limiting power to detect associations (15). This is especially true in the case of lifetime novel lysergamide use (N = 9 unweighted respondents), where all lifetime novel lysergamide users reported lifetime classic lysergamide use. It is quite possible that data from surveys with predetermined items assessing novel psychedelic use would yield different findings.

## Conclusions

The present research suggests that classic tryptamine psychedelics (i.e., ayahuasca, DMT, and psilocybin) may hold the greatest therapeutic potential of the psychedelics in that lifetime use of these substances was uniquely associated with a decreased likelihood of psychological distress and suicidal thinking. Novel phenethylamines, by contrast, might be distinct from other psychedelics in that lifetime use of these substances was independently associated with an increased likelihood of suicidal thinking and planning. Of course, the present data are by no means definitive, and it is possible that the range of psychedelic substances have clinical utility. Nevertheless, as clinical research with psychedelics remains in its infancy, the current study points to classic tryptamines as the best candidates for further study, with novel phenethylamines posing the potential for harm. Future research should aim to combine population-level methodology with chemical and pharmacological data to further investigate the therapeutic potential of classic and novel phenethylamine, tryptamine, and lysergamide psychedelics.

## Data Availability Statement

All datasets generated for this study are available on the Open Science Framework at the following link: https://osf.io/xgqmd/.

## Author Contributions

JS was the primary author who cleaned data, conducted analyses and drafted the manuscript summarizing the findings. CN contributed meaningful pharmacological expertise to inform methodology and aid in the interpretation of results. PH, corresponding author, was responsible for ensuring analyses were conducted and interpreted correctly.

## Conflict of Interest

The authors declare that the research was conducted in the absence of any commercial or financial relationships that could be construed as a potential conflict of interest.
