# Supplementary material for: Population Survey Data Informing the Therapeutic Potential of Classic and Novel Phenethylamine, Tryptamine, and Lysergamide Psychedelics
Source: Front Psychiatry. 2020 Feb 11;10:896. doi: 10.3389/fpsyt.2019.00896 (PMC7026018; doi:10.3389/fpsyt.2019.00896)
Supplement: Supplementary file 1 [file DataSheet_1.docx]

**Supplementary Table 1. Psychosocial characteristics of the total analytic sample and classic and novel phenethylamine, tryptamine, and lysergamide psychedelic users.**

| **Variable** | **Total Analytic Sample** | **Classic Phenethylamine**  **Use** | **Classic Tryptamine**  **Use** | **Classic Lysergamide Use** | **Novel Phenethylamine**  **Use** | **Novel Tryptamine**  **Use** | **Novel Lysergamide Use** |
| --- | --- | --- | --- | --- | --- | --- | --- |
|  | Weighted % | Weighted % | Weighted % | Weighted % | Weighted % | Weighted % | Weighted % |
| **Age, years** |  |  |  |  |  |  |  |
| 12 to 17 | 9.5 | 0.5 | 1.5 | 1.1 | 2.5 | 1.7 | 0.0 |
| 18 to 25 | 13.1 | 4.6 | 15.3 | 9.9 | 51.5 | 31.9 | 97.1 |
| 26 to 34 | 14.3 | 9.7 | 23.1 | 18.2 | 28.9 | 22.4 | 2.9 |
| 35 to 49 | 24 | 24.5 | 30.4 | 33.0 | 8.9 | 8.2 | 0.0 |
| 50 and older | 39.4 | 60.7 | 29.7 | 37.8 | 8.2 | 35.9 | 0.0 |
| **Sex** |  |  |  |  |  |  |  |
| Male | 48.5 | 68.3 | 66.3 | 63.2 | 78.5 | 87.7 | 100.0 |
| Female | 51.5 | 31.7 | 33.7 | 36.8 | 21.5 | 12.3 | 0.0 |
| **Ethnoracial identity** |  |  |  |  |  |  |  |
| Non-Hispanic White | 65.2 | 83.2 | 85.0 | 83.9 | 80.9 | 60.1 | 90.3 |
| Non-Hispanic African American | 11.9 | 4.3 | 2.0 | 3.8 | 0.8 | 0.0 | 0.0 |
| Non-Hispanic Native American/Alaska Native | 0.5 | 1.9 | 0.6 | 0.6 | 0.4 | 1.7 | 0.0 |
| Non-Hispanic Native Hawaiian/Pacific Islander | 0.4 | 0.2 | 0.2 | 0.2 | 0.1 | 0.0 | 0.0 |
| Non-Hispanic Asian | 4.9 | 0.7 | 1.5 | 1.2 | 1.0 | 0.0 | 0.0 |
| Non-Hispanic more than one race | 1.5 | 2.6 | 2.4 | 2.2 | 6.8 | 34.5 | 9.1 |
| Hispanic | 15.5 | 7.1 | 8.3 | 8.2 | 10.0 | 3.7 | 0.6 |
| **Educational attainment** |  |  |  |  |  |  |  |
| 5^th^ grade or less | 1.8 | 0.2 | 0.3 | 0.2 | 0.0 | 0.0 | 0.0 |
| 6^th^ grade | 2.5 | 0.2 | 0.1 | 0.2 | 0.0 | 0.0 | 0.0 |
| 7^th^ grade | 2.0 | 0.2 | 0.2 | 0.2 | 0.0 | 0.0 | 0.0 |
| 8^th^ grade | 2.9 | 1.0 | 0.9 | 1.0 | 0.2 | 0.0 | 0.0 |
| 9^th^ grade | 3.6 | 1.9 | 1.7 | 2.0 | 1.2 | 0.0 | 9.1 |
| 10^th^ grade | 3.9 | 2.8 | 2.9 | 3.2 | 1.5 | 2.6 | 8.2 |
| 11^th^ grade | 5.3 | 4.1 | 4.3 | 4.8 | 5.6 | 2.8 | 0.7 |
| 12^th^ grade | 25.9 | 27.2 | 24.1 | 27.5 | 20.9 | 15.3 | 23.7 |
| Freshman college year | 11.7 | 15.0 | 15.4 | 15.5 | 22.3 | 17.5 | 58.4 |
| Sophomore or junior college year | 13.4 | 17.5 | 16.8 | 16.8 | 21.2 | 10.0 | 0.0 |
| Senior college year or more | 27.0 | 29.8 | 33.3 | 28.6 | 27.1 | 51.8 | 0.0 |
| **Annual household income** |  |  |  |  |  |  |  |
| Less than $20,000 | 17.8 | 17.7 | 16.5 | 16.7 | 31.3 | 46.8 | 66.7 |
| $20,000 to $49,999 | 31.4 | 28.6 | 28.7 | 30.0 | 27.9 | 21.3 | 12.0 |
| $50,000 to $74,999 | 16.8 | 17.4 | 16.9 | 17.5 | 13.8 | 7.2 | 0.0 |
| $75,000 or more | 34.0 | 36.4 | 37.9 | 35.8 | 26.9 | 24.7 | 21.3 |
| **Marital Status** |  |  |  |  |  |  |  |
| Married | 50.1 | 50.4 | 43.0 | 46.6 | 15.7 | 39.7 | 0.0 |
| Divorced/separated | 13.2 | 24.1 | 16.7 | 20.3 | 3.5 | 6.1 | 0.0 |
| Widowed | 5.6 | 3.2 | 1.6 | 2.2 | 1.0 | 0.0 | 0.0 |
| Never married | 31.1 | 22.3 | 38.7 | 30.9 | 79.8 | 54.2 | 100.0 |
| **Self-reported engagement in risky behavior** |  |  |  |  |  |  |  |
| Never | 51.3 | 30.8 | 23.8 | 28.9 | 11.7 | 5.3 | 2.9 |
| Seldom | 33.4 | 42.9 | 45.3 | 43.9 | 44.6 | 65.7 | 66.7 |
| Sometimes | 12.7 | 23.5 | 27.3 | 24.1 | 36.2 | 23.0 | 22.2 |
| Always | 1.6 | 2.9 | 3.6 | 3.1 | 7.5 | 6.0 | 8.2 |
| **Lifetime illicit substance use** |  |  |  |  |  |  |  |
| Lifetime cocaine use | 14.7 | 80.1 | 74.9 | 75.2 | 84.6 | 90.6 | 93.4 |
| Lifetime other stimulant use | 7.4 | 38.5 | 37.1 | 35.4 | 67.8 | 75.9 | 84.9 |
| Lifetime sedative use | 3.7 | 28.3 | 19.0 | 19.7 | 22.5 | 12.4 | 2.9 |
| Lifetime tranquilizer use | 9.5 | 38.1 | 39.7 | 37.1 | 66.6 | 51.5 | 84.0 |
| Lifetime heroin use | 1.8 | 17.7 | 12.9 | 13.4 | 34.6 | 43.9 | 78.0 |
| Lifetime pain reliever use | 18.1 | 48.7 | 53.5 | 49.1 | 75.6 | 47.0 | 93.4 |
| Lifetime marijuana use | 43.1 | 97.8 | 98.7 | 98.5 | 99.7 | 98.6 | 100.0 |
| Lifetime PCP use | 2.4 | 31.9 | 17.8 | 21.2 | 14.6 | 12.3 | 6.3 |
| Lifetime Ecstasy use | 6.4 | 28.3 | 43.9 | 37.4 | 86.0 | 89.3 | 93.4 |
| Lifetime inhalant use | 9.6 | 46.7 | 48.4 | 45.2 | 67.5 | 75.5 | 84.9 |
|  |  |  |  |  |  |  |  |
| **Unweighted /**  **Weighted N** | 354,535 / 260,964,827 | 11,768 / 10,332,715 | 37,434 / 22,077,615 | 34,730 / 24,664,123 | 764 /  277,683 | 56 /  30,835 | 8 /  2,237 |

**Supplementary Table 2. Correlations among the six independent variables included in the regression model**

| **Variable** | **Classic Phenethylamine**  **Use** | **Classic Tryptamine**  **Use** | **Classic Lysergamide Use** | **Novel Phenethylamine**  **Use** | **Novel Tryptamine**  **Use** | **Novel Lysergamide Use** |
| --- | --- | --- | --- | --- | --- | --- |
|  |  |  |  |  |  |  |
| **Lifetime Classic Phenethylamine Use** |  |  |  |  |  |  |
|  |  |  |  |  |  |  |
| **Lifetime Classic Tryptamine Use** | 0.368 | - |  |  |  |  |
|  |  |  |  |  |  |  |
| **Lifetime Classic Lysergamide Use** | 0.42 | 0.61 | - |  |  |  |
|  |  |  |  |  |  |  |
| **Lifetime Novel Phenethylamine Use** | 0.082 | 0.12 | 0.12 | - |  |  |
|  |  |  |  |  |  |  |
| **Lifetime Novel Tryptamine Use** | 0.034 | 0.037 | 0.038 | 0.14 | - |  |
|  |  |  |  |  |  |  |
| **Lifetime Novel Lysergamide Use** | 0.008 | 0.012 | 0.014 | 0.044 | 0.039 | - |
|  |  |  |  |  |  |  |

**Note. Phi correlation coefficients are reported and statistically significant at p<0.001**
